# Identifying differentially methylated genes using mixed effect and generalized least square models

**DOI:** 10.1186/1471-2105-10-404

**Published:** 2009-12-09

**Authors:** Shuying Sun, Pearlly S Yan, Tim HM Huang, Shili Lin

**Affiliations:** 1 Case Comprehensive Cancer Center, Case Western Reserve University, Cleveland, Ohio, 44106, USA; 2 Department of Epidemiology and Biostatistics, Case Western Reserve University, Cleveland, Ohio, 44106, USA; 3 Human Cancer Genetics Program, The Ohio State University, Columbus, Ohio, 43210, USA; 4 Department of Statistics, The Ohio State University, Columbus, Ohio, 43210, USA

## Abstract

**Background:**

DNA methylation plays an important role in the process of tumorigenesis. Identifying differentially methylated genes or CpG islands (CGIs) associated with genes between two tumor subtypes is thus an important biological question. The methylation status of all CGIs in the whole genome can be assayed with differential methylation hybridization (DMH) microarrays. However, patient samples or cell lines are heterogeneous, so their methylation pattern may be very different. In addition, neighboring probes at each CGI are correlated. How these factors affect the analysis of DMH data is unknown.

**Results:**

We propose a new method for identifying differentially methylated (DM) genes by identifying the associated DM CGI(s). At each CGI, we implement four different mixed effect and generalized least square models to identify DM genes between two groups. We compare four models with a simple least square regression model to study the impact of incorporating random effects and correlations.

**Conclusions:**

We demonstrate that the inclusion (or exclusion) of random effects and the choice of correlation structures can significantly affect the results of the data analysis. We also assess the false discovery rate of different models using CGIs associated with housekeeping genes.

## Background

DNA methylation is the addition of a methyl group (CH_3_) to the 5's cytocine (C) at a CG site. It can be inherited without changing the original DNA sequences. This epigenetic modification plays an important role in regulating gene expression, and it may cause tumor suppressor gene silencing [1]. Over the last two decades, many biological and computational studies have been done to investigate the methylation patterns in different tissues. These studies either focus on candidate genes such as p16 and RASSF1A [2] or on different chromosomes [3-7]. Most of these studies focus on cancer since methylation patterns are changed in neoplasia. These changes may include regional or genome-wide gain or loss of methylation [8]. In order to gain a genome-wide understanding of how changes in methylation affect tumor growth, the DMH protocol [9-11] has been used to simultaneously assay the methylation status of all known CGIs, genomic regions rich in CG sites [12].

Previous DMH microarray studies mainly focus on identifying genes that are differentially methylated between normal individuals and cancer patients (or cell lines). They identify the genes that are hypermethylated (more methylation in cancer than normal) or hypomethylated (less methylation in cancer than normal). The data analysis of these studies mainly focuses on identifying DM genes by identifying DM probes. For example, an arbitrary log ratio cut off of 1.5 has been used [13], and a Gamma-Normal-Gamma model has been applied to identify differentially methylated probes [14]. However, a single high or low log ratio probe may not represent true biological signals due to the large impact of probe affinity. This is because the intensity of each probe is related to its sequence, and different microarray probes may have similar sequences. Therefore, both specific and non-specific binding could occur. With non-specific binding, two probes against the same region (e.g., a short CGI) may have very different intensity values. This issue has been well known and has been addressed in the context of gene expression microarrays [15-18]. In addition to probe affinity, other factors such as the polymerase chain reaction (PCR) application effect, sample preparations, and the sensitivity of scanners will also affect probe intensities [17]. Furthermore, it has been shown that neighboring probes are highly correlated over hundreds of bases [3]. As a result, we can not assume that all probes are independent. In addition, because cancer patients or cell lines may have different levels of methylation signals, it is important to consider random effects in the model too.

Unlike previous DMH studies, this paper focuses on identifying genes that are differentially methylated between two tumor subtypes (or two racial groups) rather than between normal and cancerous cells. We propose a novel method for identifying a DM gene by pooling all probes in its associated CGI together and incorporating the correlation structures for probes in the same CGI. To implement this method, we apply two mixed effect models and two generalized least square models to incorporate the heterogeneity of different arrays (cell lines) and study the correlation structures between probes. We compare the results of these four models with the ones obtained from a simple least square regression model and find that it is important to incorporate the random effect and choose a correlation structure properly.

## Methods

### DMH microarray protocol, data preprocessing and description

Microarray technology has brought about a revolution in our understanding of normal and abnormal molecular processes. With the aid of this technology, it is now possible to identify DNA methylation patterns in specific regions of chromosomes or even in the entire genome. The DMH protocol [9-11] utilizes restriction enzymes to reduce the complexity of the genome while preserving GC-rich regions (many of which fall in and around CGIs) for methylation profiling. A brief outline of the DMH protocol is described below:

#### Step 1

Genomic DNA samples are sonicated into 400-500 bp fragments, and linkers are ligated to these fragments.

#### Step 2

The methylation status of the genome of interest can be investigated by methylation sensitive restriction enzymes. In this particular study, the investigation is achieved with two methylation sensitive enzymes, HpaII and HinPI, which have the recognition sites of CCGG and CGCG, respectively. These restriction enzymes will cut all of these CG-containing sites if the Cs are not methylated. These enzymes will therefore remove all linker-ligated fragments containing unmethylated CG dinucleotides, leaving behind fragments that are 100% methylated or those that have no HpaII and HinPI recognition sites.

#### Step 3

The DNA fragments that are not restricted are then enriched with the polymerase chain reaction (PCR) to provide sufficient materials for fluorescent labeling and microarray hybridization. The DMH protocol is applied to both test samples (e.g., cancer patient material or cancer cell lines) and control samples (common normal reference or paired normal tissue). Both types of samples are labeled with their respective fluorescent dyes: Cy5 dye pseudo-colored as red for test samples and Cy3 dye pseudo-colored as green for control samples. They are then combined in equal quantity and competitively hybridized to a prepared microarray slide, in particular, an Agilent 244 K human CGI array.

In total, there are about 237,220 probes that span 25,000 CGIs found in the human genome. The majority (about 75%) of CGIs are covered by at most 10 probes. This information is summarized in Table 1. There is one probe within every 100 bp region per CGI, and the length of probe sequences ranges from 45 to 60 bp. For each probe, we use the base two log ratio of red over green intensity, log_2_(Cy5/Cy3), as the observed methylation signal. Large positive log ratios show that there are more methylations in test samples (e.g., cancer patients) than in control samples (e.g. normal individuals). This means that there are strong hypermethylation signals in test samples. In contrast, large negative log ratios indicate that there are less methylations in test than in control samples, implying that there are strong hypomethylation signals in test samples. Several consecutive probes are expected to have similar positive (or negative) log ratios because the DNA fragments that are hybridized to the array are about 400-500 bp.

**Table 1:** Summary of probe numbers and CGIs.

# of probes	1~2	3~10	11~15	16~20	21~30	> 30
count	2753	16580	3323	1190	721	358

Proportion	0.11	0.67	0.13	0.05	0.03	0.01

List of the count and proportion of all 24925 CGIs that have 1 to 2, 3 to 10, 11 to 15, 16 to 20, 21 to 30, and more than 30 probes.

In our example data, 38 breast cancer cell line microarrays are divided into two tumor subtypes, basal-like and luminal types [19]. Each group has 19 arrays. For each array, the background correction is done using the "edwards method" [20]. Dye effects are corrected using the standard within array loess normalization in the Bioconductor package "limma" [21]. The between array normalization is done using the quantile method [22,23].

**Figure 1:**
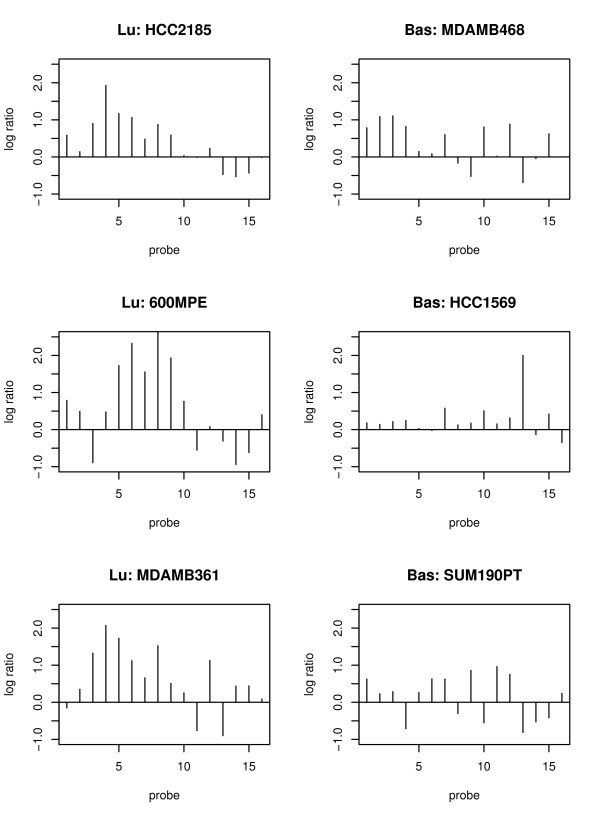
**The normalized data of a 16 probe-CGI across tumor subtypes**. The normalized data of 16 probes at one CGI (associated with FOXD2 gene) for 6 cell lines. The left three plots with "Lu:" in the title of each plot are for luminal cell lines. The right three plots with "Bas:" in the title of each plot are for basal-like cell lines. The y-axis is the log ratio of methylation signals at each probe. The x-axis is the probe index at each CGI. Probes are ordered according to their physical locations, and the distance between two consecutive probes is roughly 50 bp.

In order to demonstrate the heterogeneous methylation patterns in different tumor subtypes and the variability in cell lines, we plot the normalized data of one CGI associated with the gene FOXD2 as shown in Figure 1. In this figure, the probes are ordered according to their physical location. The left three plots are for luminal cell lines, and the right three plots are for basal-like ones. Neighboring probes in each cell line tend to have similar methylation signals. Cell lines belonging to the same tumor subtypes have similar methylation patterns. For example, the methylation signals of the three luminal cell lines are stronger than the ones in the basal-like subtype. Our goal is to identify those CGIs (and their associated genes) that are differentially methylated between two tumor subtypes.

### Statistical models

In order to identify differentially methylated genes between two tumor subtypes, we propose the idea of identifying differentially methylated CGIs associated with them. That is, we build models to identify differentially methylated CGIs, then determine whether a gene is methylated (either at its promoter region or inside of the gene) by examining if there is any significant methylation difference at the CGI(s) associated with this gene. This method is different from the others that identify differentially methylated genes by focusing on identifying differentially methylated probes. Instead, we put the information of all probes at each CGI together to conduct analysis using different mixed effect and generalized least square models.

A mixed effect model includes fixed effect(s) and random effect(s). A fixed effect is usually a well-defined population factor with finite levels. We are interested in estimating different levels of a fixed effect and testing to see if they are significantly different. For example, in our DMH data the tumor subtype factor, which has two levels, basal-like and luminal types, is a fixed effect and we want to test to see if the methylation levels of these two subtypes are significantly different from each other. A random effect is related to some individual experiment units that are selected from a larger (potentially infinite) underlying population and represent a certain amount of random variation. For example, the cell lines (or arrays) within each subgroup can be thought of as random samples from their representative population. However, we are not particularly interested in the differences between the cell lines within the same population. As such, a random effect component is included to model the variability. The inclusion of a random effect can help us generalize the analysis result to the whole population.

Furthermore, each cell line can be considered as a "block" of probes, which will allow us to investigate the correlation structure between probes within the same array. Alternatively, a generalized least square model can also be used to study correlations among probes of a CGI within the same cell line/array. Various models may be used to study such correlations. In this paper, we apply two commonly used structures. One is "compound-symmetry" correlation; that is, all within-group errors are assumed to have equal correlations. Another one is based on the autoregressive model in which we assume data points are observed at integer time (or location) points, and the current observation is a linear function of previous ones and an error term [24].

Although we know neighboring probes are correlated, it is still unclear what specific correlation structure is the best fit. In addition, it is not clear how the random variation among different cell lines (in the same tumor subtype) will affect the analysis results. Therefore, we explore the following four models: (1) the mixed effect model with the array effect as a random effect, M1 (mix); (2) the mixed effect model with the array effect as a random effect and the probe correlation structure modeled using an autoregressive model of order one (AR1), M2 (mix.AR1); (3) the generalized least square model with a compound symmetry correlation structure, M3 (gls.comSym); and (4) the generalized least square model with an AR1 model of correlation structure, M4 (gls.AR1).

In the following four models, we use *g *to represent the group index with *g *= 1 and 2 corresponding to tumor subtypes: basal-like and luminal types. Likewise, *p *is the probe index (*p *= 1, ..., P, where P is the total number of probes in one CGI). *a *is the array (or cell line) index (*a *= 1, 2, ..., N, where N is the total number of arrays). *α*_*g *_and *β*_*p *_represent the group and probe effects, and they are fixed effects. *γ*_*a *_is the random effect. *ε*_*gpa *_is the residual error. *γ*_*a *_and *ε*_*gpa *_are independent in all models.

(1). M1 (mix): it is the mixed effect model,

*y*_*gpa *_= *α*_*g *_+ *β*_*p *_+ *γ*_*a *_+ *ε*_*gpa*_,  for any *a*, and  for any *g*, *p *and *a*. The correlation structure is

Therefore, the correlation between any two different probes (*p*_1 _and *p*) at each CGI is .

(2). M2 (mix.AR1): it is the mixed effect model with an AR1 correlation structure, *y*_*gpa *_= *α*_*g *_+ *β*_*p *_+ *γ*_*a *_+ *ε*_*gpa*_,  for any *a*, and  for any *g*, *p *and *a*.

The AR1 model is used for the correlation structure of residual errors, so that

Therefore, the correlation between any two different probes (*p*_1 _and *p*) at each CGI is , where *ρ *is the correlation between two consecutive residual errors, that is,

(3). M3 (gls.comSym): it is the generalized least square model with a compound symmetry correlation structure: *y*_*gpa *_= *α*_*g *_+ *β*_*p *_+ *ε*_*gpa*_,  for any *g*, *p *and *a*. The correlation structure is

Therefore, the correlation between any two different probes (*p*_1 _and *p*) at each CGI is *ρ*_3_.

(4). M4 (gls.AR1): it is the generalized least square model with an AR1 correlation structure: *y*_*gpa *_= *α*_*g *_+ *β*_*p *_+ *ε*_*gpa*_,  for any *g*, *p *and *a*. The correlation structure is

Therefore, the correlation between any two different probes (*p*_1 _and *p*) at each CGI is .

For the above models, we are interested in testing the null hypothesis (H_0_) that there is no methylation difference between the two tumor subtypes, i.e., *α*_1 _= *α*_2_, at each CGI. If the test p-value of one CGI is less than a certain threshold, then it is indicative of strong evidence that this CGI and its associated gene(s) are differentially methylated between two tumor subtypes. All the above mixed effect models and generalized least square models are implemented for each CGI using the R package "nlme" [24].

## Results

### Comparisons of different models

**Figure 2:**
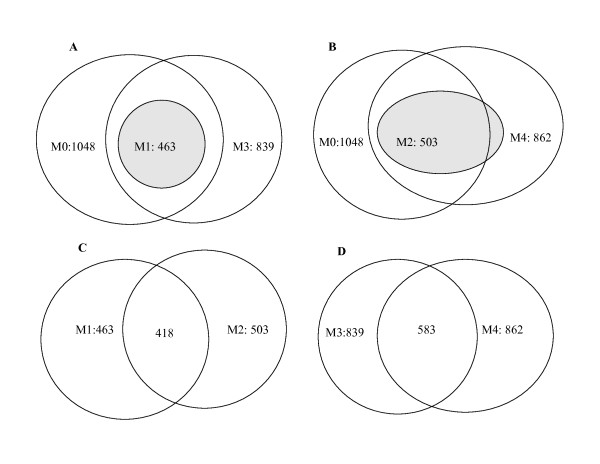
**Overlaps of CGIs obtained from different models**. Each plot is a set of Venn Diagrams that shows the overlap of CGIs selected by different models. (A) is for models M0, M1 and M3. (B) is for models M0, M2 and M4. (C) is for models M1 and M2. (D) is for models M3 and M4.

**Figure 3:**
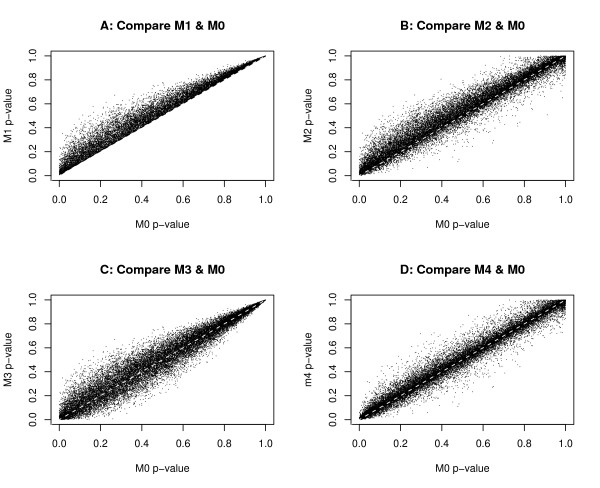
**The plots of p-values of all CGIs**. The plots of p-values of all CGIs obtained from models M1, M2, M3 and M4 against the p-values from the linear model M0: (A) M1 versus M0, (B) M2 versus M0, (C) M3 versus M0, and (D) M4 versus M0. The white dashed line is the straight line with an intercept of 0 and a slope of 1.

In order to see the impact of using different correlation structures and including or excluding random effects, we compare the above four models with the simple least square regression model M0 (lm), which does not incorporate any correlation structure and only has fixed effects, that is, *y*_*gpa *_= *α*_*g *_+ *β*_*p *_+ *ε*_*gpa*_,  for any *g*, *p *and *a*. The test results of all five models, that is, the numbers of CGIs with p-values < 0.05, are summarized in Table 2. The overlaps of these models are shown in Figure 2. In addition, we also plot the p-values of all CGIs from four models (M1, M2, M3 and M4) against the p-values of the model M0, which does not incorporate correlations between probes (see Figure 3). The two top plots of this figure are the models with random effects included (models M1 and M2). The model M1 has larger p-values than M0 at *all *CGIs, while the model M2 has larger p-values than M0 at *most *CGIs. The two bottom plots of Figure 3 are the models without any random effect included. These plots show that, compared with the linear model (M0), models M3 and M4 have some CGIs with larger p-values and some with smaller ones than M0.

**Table 2:** The number of CGIs with p-values < 0.05 in five models.

Models	M1	M2	M3	M4	M0
# of CGI with p < 0.05	463	503	839	862	1048

The mixed effect models (M1 and M2) identify roughly half as many DM CGIs as identified by the non-random effect models (M3, M4 and M0). This could be due to the fact that these mixed effect models include the random effect to incorporate the variation of different cell line samples. When the random effect is included, a positive correlation structure is introduced to the model. Therefore, we tend to get larger p-values. This is useful in reducing bias due to the noise, which could be caused by many unmeasured factors, and may introduce improper negative correlations that may not be consistent with the underlying methylation pattern and the DMH microarray protocol.

**Figure 4:**
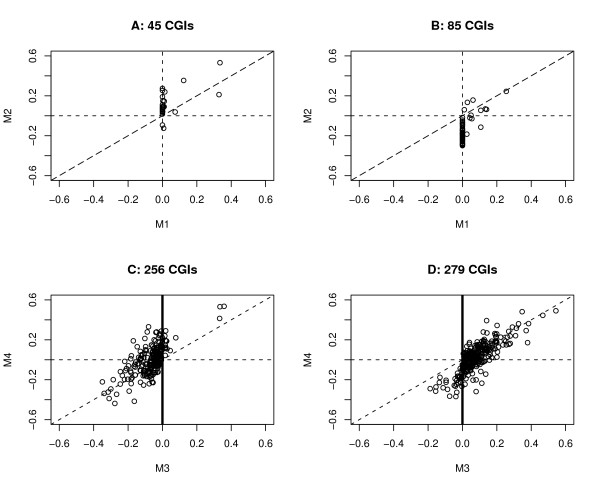
**Correlation estimate comparison plot**. Plots of correlation estimates for CGIs that are selected by one model but not by another one: (A) 45 CGIs by M1 but not by M2, (B) 85 CGIs by M2 but not by M1, (C) 256 CGIs by M3 but not by M4, and (D) 279 CGIs by M4 but not by M3.

Because the mixed effect models appear to be better suited for the purpose of analyzing CGI methylation data, we focus on further comparing M1 with M2. We find that 45 CGIs are obtained from the model M1 but not from M2, the majority (30/45 = 67%) of which have correlations equal to zero in the M1 model and most of which (except 2/45 CGIs) have positive correlation estimates in M2; for those 85 CGIs that are selected by the model M2 but not by M1, the majority (72/85 = 85%) have correlations equal to zero in the M1 model, and most of those 85 CGIs (except 8/85 of them) have negative correlations in model M2 (see the top two plots in Figure 4).

### Relationship between correlation estimates and the number of probes

**Figure 5:**
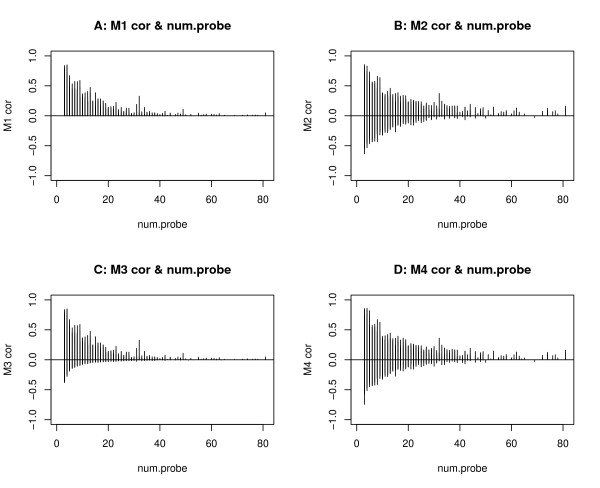
**Plots of correlation estimates and number of probes**. Plots of the correlation estimates obtained from different models (M1 - M4) against the number of probes: (A) model M1, (B) model M2, (C) model M3, and (D) model M4. In each plot, the horizontal axis is the number of probes and the vertical axis is the correlation estimates obtained from each model.

In the previous section we explore and compare the results and features of those four different models (M1, M2, M3 and M4) using the linear model M0 as the base line. The comparison results show that the p-values are related to the correlation structures (estimates). As far as the correlation between probes is concerned, our understanding about the microarray protocol is that this might be related to the number of probes at each CGI. According to the DMH protocol, several consecutive probes (across several hundred bases) in the same CGI are meant to have similar log ratios; that is, they are all positive (or negative) and have similar values. However, for much longer CGIs with many probes, the methylation pattern can vary along the CGI; that is, there could be very strong methylation signals in one end of the CGI, but not many signals in the other end. For the shorter CGIs with only 3 or 4 probes, the methylation signals of these probes are supposed to be similar. However, our exploration of the data shows that this may not be true due to the noise of the data. In order to fully understand the relationship between the correlation estimate and number of probes at each CGI, we make plots for all CGIs as shown in Figure 5.

In Figure 5, "A" is the correlation estimates of the model M1, that is, the estimates of the intra-class correlation, , for all CGIs. These estimates are only related to the variances of the random effect and the residual error, so they are all positive. "B" is the correlation estimates (*ρ*_2_) of the model M2 for all CGIs, some of which have positive correlations, while the others have negative ones. "C" is the correlation estimates of the model M3 for all CGIs, the majority of which are positive. "D" is the correlation estimates of the model M4 for all CGIs. About half of these estimates are positive and half are negative.

**Figure 6:**
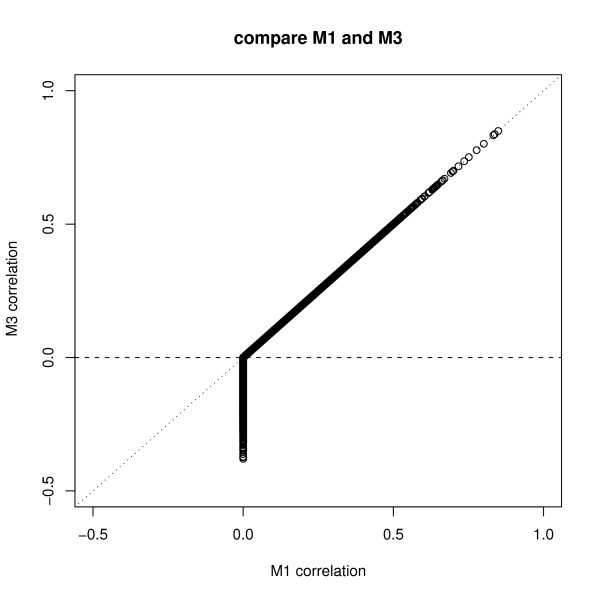
**Correlation estimates of M1 and M3**. This plot compares the correlation estimates obtained from models M1 and M3. 12080 CGIs, corresponding to the points above the horizontal line, have positive correlations in both M1 and M3. 9210 CGIs, corresponding to the points blow the horizontal line, have negative correlations in only M3.

Figure 5 shows that the CGIs with a small number of probes tend to have very large positive or negative correlations. Large positive correlation estimates could be more consistent with the underlying methylation pattern and the DMH design, while large negative correlations could be due to some artificial effects. In order to understand the correlation pattern better, we compare the correlation estimates of models M1 and M3 since these two models have similar correlation structures. The correlation estimates of these two models are plotted in Figure 6. In this comparison, we find that 12080 CGIs have positive correlations in both models M1 and M3. 9210 CGIs have negative correlations in M3, but their correlations are equal to 0 in the model M1.

**Figure 7:**
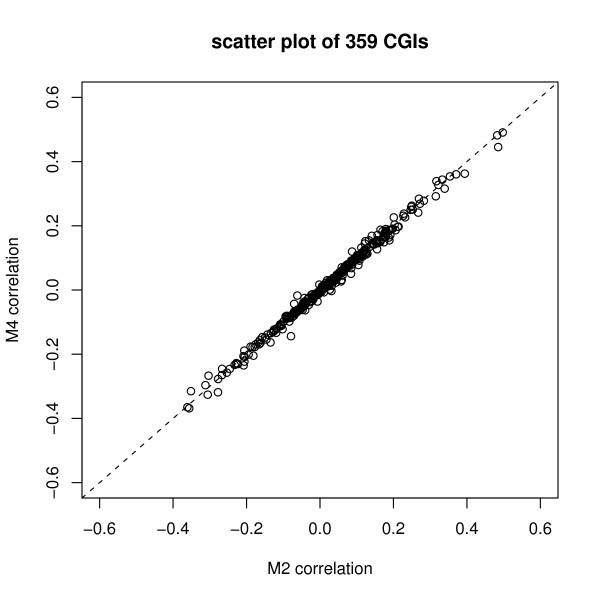
**Correlation estimates of CGIs obtained from model M4 but not M2**. This plot compares the correlation estimates of 359 CGIs that are obtained from model M4 but not M2. That is, their M4 model p-value is less than 0.05, but their M2 model p-value is larger than 0.05.

In order to explore whether the negative or positive correlation estimates have a major impact on the final conclusion of the significant differentially methylated CGIs, we study the CGIs with p < 0.05 in models M1 and M3, that is, those 463 CGIs with p < 0.05 in model M1 (mix) and those 839 CGIs with p < 0.05 in model M3 (gls.comSym). In fact, those 463 CGIs from M1 are part of those 839 CGIs. For the remainder of those 376 CGIs, 85% (319/376) of them have negative correlation; that is, the 376 small p-values from model M3 are mainly due to the negative correlations. Besides the comparisons of models M1 and M3, we also compare models M2 and M4. There are 503 CGIs with p < 0.05 in M2 (mix.AR1), and 862 CGIs with p < 0.05 in M4 (gls.AR1). Among these CGIs, 503 CGIs from model M2 are in fact part of those 862 CGIs from M4. They have p < 0.05 in both models M2 and M4. 359 CGIs have p < 0.05 only in the M4 (gls.AR1) model, but not in M2 (mix.AR1). Figure 7 shows that, for those 359 CGIs, the correlation estimates from M4 are similar to the ones from M2. However, these 359 CGIs have much larger p-values in the model M2 than in M4. This may be due to the fact that microarray samples are heterogeneous and the model M4 does not include random effects.

### Assessing false discovery rate

**Figure 8:**
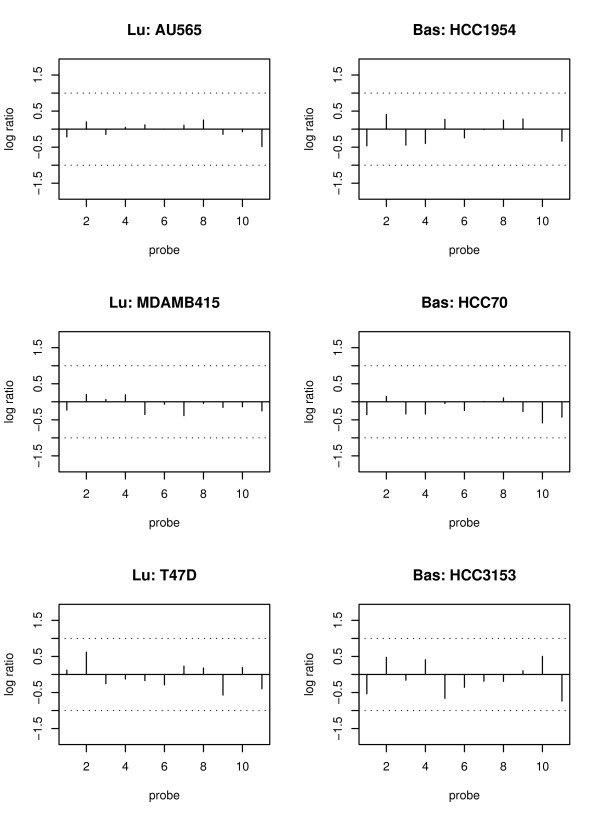
**The normalized data of the housekeeping gene RAD9A**. The normalized data of 11 probes at one CGI (associated with RAD9A gene) for 6 cell lines. The left three plots with "Lu:" in the title of each plot are for luminal cell lines. The right three plots with "Bas:" in the title of each plot are for basal-like cell lines. The y-axis is the log ratio of methylation signals at each probe. The x-axis is the probe index at each CGI. Probes are ordered according to their physical locations, and the distance between two consecutive probes is roughly 50 bp.

As shown in the previous section, the inclusion (or exclusion) of random effects and the choice of correlation structure can greatly affect the results. Therefore, it is very important to access the false discovery rate (or specificity) of all models. In order to do so, we borrow some information from housekeeping (HK) genes and treat them as known "non-differentially methylated genes". This is because HK genes are known to maintain the basic function of a cell and are relatively stable, so their methylation signals are not supposed to be very different between two groups. Figure 8 shows the methylation pattern of a HK gene, where the log ratios of most probes in all six cell lines (three in each tumor subtype) are close to zero. In this paper, we use 205 HK genes selected from 575 such genes [25]. These 205 genes are associated with only one CGI and there is at least one promoter probe. The results of comparing the five models using 205 HK genes are listed in Table 3. This table shows that the mixed effect models (i.e., M1 and M2) identify fewer HK genes. In particular, the number of DM HK genes obtained from the model M1 is half as many as that identified by models M4 and M0, leading to a smaller false discovery rate. This result is consistent with the one shown in Table 2; that is, the number of CGIs identified by non-random effect models (especially the model M0) is about twice as large as the one identified by the model M1. These results suggest that mixed effect models may be more appropriate for modelling the variability among cell lines within the same group compared to the other models.

**Table 3:** The number of housekeeping genes with p < 0.05 in five models.

Models	M1	M2	M3	M4	M0
P < 0.05	6	8	9	12	11

## Discussion and Conclusions

We propose a new method for identifying differentially methylated genes by identifying differentially methylated CGIs. With this method, we can consider the information of all probes in each CGI as a whole. One advantage of this method is that we can add a "probe" factor to account for the noise and variation due to many unmeasured factors (e.g., effects due to PCR and human error). Another advantage is that we can model the correlation between probes in the same array at each CGI. Our comparison results do show that this is necessary as the correlations between probes can affect the test results significantly.

Because cancer cell lines or patient samples are in general heterogeneous, they may have very different levels of methylation. Hence, the inclusion (or exclusion) of random effects is crucial in the data analysis. Our assessment of the false discovery rate using HK genes also shows that the mixed effect models (M1 and M2) have relatively smaller false discover rates. Therefore, we recommend using a mixed effect model instead of a generalized least square model. As for whether to use M1 or M2, we recommend using the model M1 for the following two reasons. First, according to the DMH protocol, the consecutive probes are supposed to be positively correlated. Second, the AR1 model could be too specific for this type of noisy and complex data; that is, 'forcing' a specific model like AR1 to identify the local correlation may not be proper. As for the selected candidate CGIs (genes), we recommend ignoring short CGIs that have only three or four probes and have zero correlation estimates or investigating them more carefully. We suggest this since methylation signals should be very similar within 300-400 bases (covering about three or four probes) and such a zero correlation estimate is more than likely due to noise.

As for the sensitivity, it is very important to access this with genes that are known to be differentially methylated between two tumor subtypes. Currently, we do not have such a list of genes. Instead, we make use of some known biological information about methylation patterns [3] and the DMH protocol [9-11] to make the above suggestions. One key assumption of linear models is normality. In order to validate the assumption, we used the Kolmogrov-Smironov (KS) test on the residuals of the M1 (mix) model. After the "BH" multiple test correction [26], no CGIs fail the KS test. Therefore, it is reasonable to assume the normal distribution.

After the within-array and between-array normalization, outlying probes are selected and deleted if there is at least one observation falling out of this range [Q1 - k*(Q3-Q1), Q3 + k*(Q3-Q1)], where k = 5, Q1 and Q3 are the 25% and 75% quantiles of the log ratios of all probes in one CGI. Note that we choose k = 5, which is larger than the usual inter quartile range (k = 1.5). With k = 5, we mean to delete those probes that have outlying observations due to noise but retain those with certain variations for further modeling.

The main focus of this paper is to address the importance of incorporating random effects and correlations in our statistical models and to see how this affects the analysis results. Therefore, the numbers of DM CGIs presented in Table 2 are the summary based on the raw p-values, and we have not done any multiple test correction. If we were to conduct multiple test corrections, especially using a stringent method, we expected that not many CGIs would be selected any more. This could be due to two reasons. First, the underlying biological truth is that the effect size is too small to survive any multiple test corrections. Second, the sample size may not be large enough to separate two tumor subtypes. For example, it has been shown that among 194 cancer patients, only about 1/6 ~1/5 of them have stronger methylation signals than normal individuals at the CST6 gene transcription start site (see Figure 7 (A) of [27]). Generally speaking, two types of cancerous cells are much more similar than cancerous and normal cells, so many more samples might be needed to really separate the methylation patterns of two tumor subtypes.

In this paper, we implement four models (M1, M2, M3 and M4) in all 22,000 CGIs, which have at least three probes. For each CGI, a p-value is obtained at each model to indicate whether there is a significant methylation difference between two tumor subtypes. The p-values for long CGIs (e.g., the ones with more than 20 probes) may not have the same meaning as the ones for the shorter CGIs (e.g., with less than 10 probes). We also discover that the long CGIs easily fail the KS normality test (i.e., raw p-value < 0.05) and have relatively smaller R^2^. Therefore, we recommend utilizing the following two methods to deal with long CGIs. First, break the long CGIs into a few parts with each part consisting of several probes (e.g., around 10 probes). Second, fit the linear model to probes at each CGI using only the probes that are located in the promoter or first exon region of certain genes, and if necessary, divide them into small parts. There are only a small percentage of CGIs (about 4%) with more than 20 probes, so this will not affect our conclusion about the relationship between correlation estimates and the number of probes.

There are other HK gene lists provided by some recent papers [28,29]. After comparing the lists from these papers with the one we used [25], we found our HK gene list is still a better choice for our purpose because it has the following two advantages. First, it is obtained using relatively a larger number of tissues than the ones used in [28,29]. Second, the microarray data set used in [25] has replicates which helps reduce some measurement noise, making results more trustworthy.

The overlap of those significantly differentially methylated CGIs obtained from all five models is in fact the overlap of the CGIs from models M1 and M2, which are 418 CGIs. These CGIs are associated with 355 genes with known annotations. Ten genes sets (pathways) are enriched using the Molecular Signature Database (MSigDB) to conduct the gene set overlapping analysis http://www.broad.mit.edu/gsea/ (see Table 4 for the pathways and the genes that overlaps with these pathways). Several of these pathways are also identified by other breast cancer gene expression analysis methods that incorporate the pathway information [30,31]. This indicates that the methylation signatures of the genes in these ten pathways could play a very important role in breast cancer studies. In addition, six genes (PIK3R2, KDR2, EPOR, MPL, BMP2, and PPP1CB) are enriched in at least two pathways. Five of these six genes are closely related to various cancers. In particular, PIK3R2, which is enriched in four pathways, belongs to the PIK3 gene family that plays an important regulatory role in various cancer related signalling pathways [32]. It has recently been identified as a significant negative prognosis factor for ovarian cancer [33]. KDR, also known as VEGFR2 gene, is important in tumor angiogenesis [34]. Due to its crucial role in angiogenesis, KDR could be used as the molecular target of early diagnosis and treatment in cancer [35,36]. EPOR is the receptor of the recombinant human erythropoietin (Epo) that is used to treat tumor related anemia [37]. The MPL mutation occurs in acute megakaryoblastic leukemia [38]. Aberrant BMP2 promoter CGI methylation has been found in both colon cancer and gastric cell lines, and the resultant loss of BMP2 expression may be related to gastric carcinogenesis [39].

**Table 4:** Gene set enrichment analysis

Gene sets	p-value	Genes in pathways
Neuroactive ligand receptor interaction	2.13 e-5	EDNRB, GABBR1, GRIK2

Cytokine cytokine receptor interaction	3.1 e-4	KDR, EPOR, MPL, TGFB2, BMP2

Regulation of actin cytoskeleton	9.21 e-4	PIK3R2, PPP1CB, LIMK2, ARPC1B

Focal adhesion	4.81 e-3	KDR, PIK3R2, PPP1CB, COL2A1, COL5A1

Natural killer cell mediated cytotoxicity	4.97 e-3	PIK3R2, NFATC3

Jak-STAT signaling pathway	5.63 e-3	EPOR, MPL, PIK3R2

Oxidative phosphorylation	6.17 e-3	ATP5A1, ATP12A

Smooth muscle contraction	6.53 e-3	PLCB3, CALM1, PRKCE

Purine metabolism	8.35 e-3	POLA1, ADA, IMPGH2

MAPK signaling pathway	1.14 e-2	MAP3K7, CACNA1C, CACNA1H, MKNK1, NTRK1, MAPKIP2, CACNG8, RASGRF2, TGFB2

CGIs could cover different parts of genes, and CGIs from promoter regions may differ from those found in the gene body with respect to their methylation levels and neighboring probe correlation levels. However, this does not affect the performance of our models since they are completely general and analyze each CGI *separately*. Therefore, they can accommodate the methylation level differences and probe correlation strength for each CGI. After the analysis is done, we can do further bioinformatic studies for the CGIs we identified. For example, we can investigate the relationship between CGI locations and probe correlation estimates. Studying the genes identified by the model M1, we found that 45% of promoter CGIs have a positive correlation estimate, and 55% of non-promoter CGIs have a positive correlation. This may indicate that the probe correlation estimates are location dependent.

Although we have only presented the implementation and comparisons of the models in one data set in this paper to focus our discussion, in fact, we have done the same analysis and comparison on an endometrial cancer data set of two racial groups (African Americans and Caucasians) and got the same conclusions. In addition, although the results of this paper are mainly for the DMH microarray data, the methods could be applied to data generated by the other methylation microarray protocols such as Methylated DNA Immunoprecipitation (MeDIP) assay.

## Authors' contributions

SS developed and implemented the models, performed all statistical analyses, drafted and revised the manuscript. PSY was involved in the data collection and helped in preparation of the manuscript. THMH oversaw the project and revised the manuscript. SL provided suggestions on the project and revised the manuscript. All authors have read and approved the final document.
